# Papillary Tumor of the Pineal Region: MR Signal Intensity Correlated to Histopathology

**DOI:** 10.1155/2015/315095

**Published:** 2015-01-22

**Authors:** Marcos Rosa Junior, Antonio Jose da Rocha, Adriano Zanon da Silva, Sergio Rosemberg

**Affiliations:** ^1^Section of Neuroradiology, Santa Casa de Misericórdia de São Paulo, São Paulo, SP, Brazil; ^2^Centro de Ciências da Saúde, Maruípe, Universidade Federal do Espírito Santo (UFES), 29043-900 Vitória, ES, Brazil; ^3^Section of Pathology, Santa Casa de Misericórdia de São Paulo, São Paulo, SP, Brazil

## Abstract

Tumors of the pineal region are rare and can be challenging to differentiate by imaging. Papillary tumor of the pineal region (PTPR) was recently recognized as a neoplasm in the World Health Organization (WHO) 2007 classification, arising from specialized ependymocytes in the subcommissural organ, which is located in the pineal region. It is a rare histological type of pineal tumor with only a few cases reported. Here, we describe a case of histologically confirmed PTPR in a 17-year-old man who presented with a headache. A literature review was performed to clarify the clinical, radiological, and pathological features of PTPR. Pineal neoplasms do not have pathognomonic imaging findings; however, we discuss T1 hyperintensity, which is a key for imaging diagnosis according to recent reports. In particular, if the hyperintensity in T1 is not due to fat, calcification, melanin, or hemorrhage in a mass of the posterior commissure or pineal region, the diagnosis of a PTPR may be suggested, as observed in this case.

## 1. Introduction

Tumors of the pineal region are rare and can be challenging to differentiate by imaging. Pineal region neoplasms occur more frequently in children, accounting for 3–8% of intracranial neoplasms in this population. They represent less than 1% of intracranial tumors in adults [1]. Tumors of the pineal gland are categorized into germ cell tumors and tumors of the pineal parenchymal. Tumors may also arise from the cell types in the proximity of the pineal gland (tumors of the pineal region). These include mainly astrocytomas and meningiomas. Papillary tumor of the pineal region (PTPR) was recently recognized as a neoplasm in the World Health Organization (WHO) 2007 classification, arising from specialized ependymocytes in the subcommissural organ, which is located in the pineal region. It is a rare type of pineal region tumor with only a few cases reported [2]. Here, we present a patient who underwent brain computed tomography, magnetic resonance (MR) imaging, and surgical resection of the tumor in the posterior commissure and pineal region, which was confirmed by pathology as PTPR.

## 2. Case Presentation

A 17-year-old man was taken to the emergency department with a severe headache. Imaging brain studies included computed tomography, with no intravenous iodinated contrast, and MR acquisition, including 3D sagittal fluid attenuated inversion recovery (FLAIR) sequence (TR 7,000 ms, TE 276 ms, and TI 2,300 ms), multiplanar T1-weighted spin-echo (T1 SE) acquisitions (TR 450 ms, TE 15 ms) before and after a single injection (0.1 mmol/kg) of intravenous dimeglumine gadopentetate (Gd), and a nonenhanced T1 spin-echo/magnetization transfer contrast (SE/MTC: TR 600 ms, TE 12 ms/magnetization transfer contrast medium pulse on resonance) sequence.

**Figure 1:**
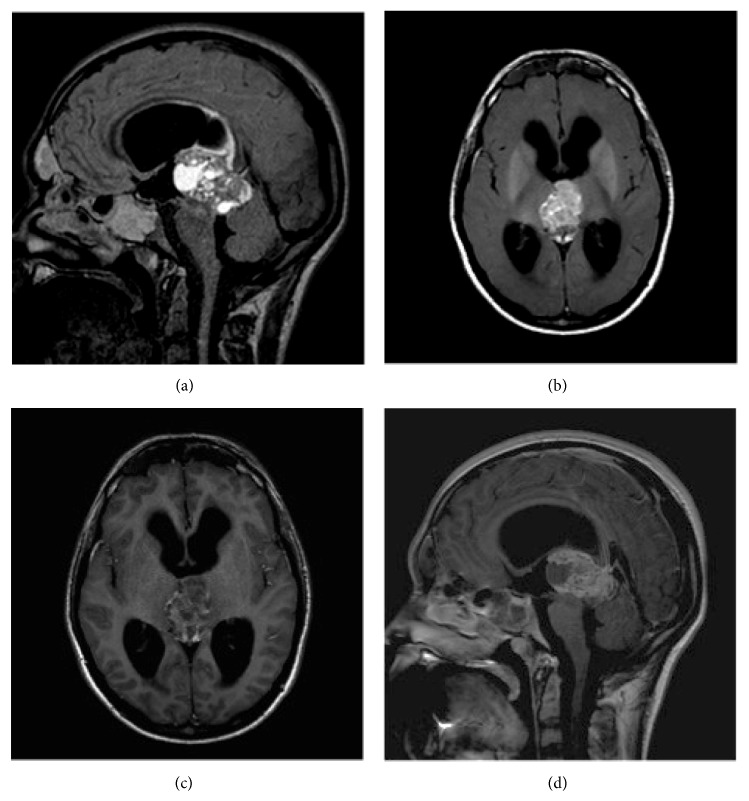
Sagittal FLAIR (a) shows solid and cystic portions, both containing high protein content. Axial T1 SE/MTC (b) shows more pronounced hyperintensity than the comparative T1 SE (c). T1 SE after gadolinium administration (d) confirms heterogeneous enhancement in the solid portion of the tumor and the cystic walls.

Imaging analysis showed a hydrocephalus caused by a tumor centered between the posterior commissure and pineal region, which compressed the tectum and mesencephalic aqueduct. Small cystic areas were noted into the mass, and the solid portions of the tumor were enhanced heterogeneously after gadolinium administration. During evaluation of this solid portion, we noticed a pronounced hyperintensity of the T1 SE/MTC over the other T1 SE images (Figure 1). Susceptibility weighted imaging (SWI) sequence confirmed small foci of hemorrhage within the tumor. There was no calcification or fat on the computed tomography (CT). The serum *α*-fetoprotein was 1.3 ng/mL (reference < 8 ng/mL), and *β*-hCG was undetectable.

## 3. Discussion

Pineal parenchymal tumors are considered to be rare lesions, accounting for less than 0.2% of intracranial neoplasms [3]. Primary tumors of the pineal region include pineal parenchymal neoplasms, germ cell tumors (GCT), and tumors arising from adjacent structures, including meningiomas, astrocytomas, and ependymomas. Metastatic lesions may also occur in this region. Approximately 40% of the pineal tumors represent GCT, whereas pineal parenchymal tumors represent approximately 27% [1]. Pineal parenchymal tumors include the low-grade pineocytoma, the intermediate-grade pineal parenchymal tumor of intermediate differentiation, and the highly malignant pineoblastoma. The WHO classification divides primary tumors of the pineal region into germinomas and nongerminomatous GCTs. Nongerminomatous GCTs include teratomas, yolk sac tumors, embryonal carcinomas, choriocarcinomas, and mixed GCTs.

**Figure 2:**
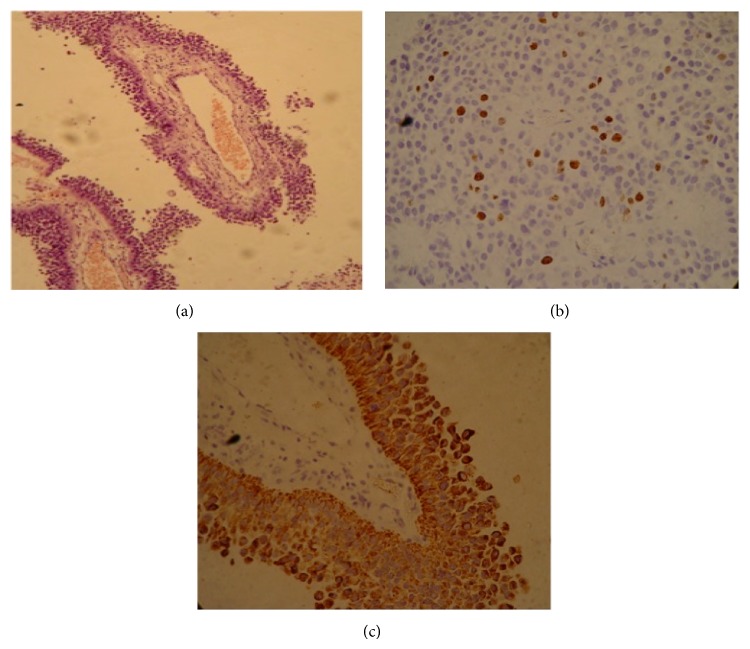
Histology of the tumor showing papillary projections (a) based on hematoxylin eosin (HE) staining. Immunohistochemistry showing 20% positive Ki67 (b) and the high expression of cytokeratin, an immunophenotype characteristic of PTPR (c).

The name PTPR is derived from the pathological description manifested by papillary features, rosettes, and pseudorosettes [4]. The neuropathology literature defines the morphologic features and immunophenotypic profiles that distinguish PTPR from the other papillary-type masses that occur in the pineal region [5]. All tumors exhibited an immunophenotype characteristic of PTPR, with high expression of cytokeratin, widespread immunoreactivity for neuron-specific enolase and the S-100 protein, focal immunoreactivity for vimentin, and the complete absence of immunoreactivity for glial fibrillary acidic protein (GFAP) (Figure 2). PTPR was recognized as a neoplasm by the WHO in 2007, corresponding to grade II or III [1]. It is assumed that PTPR arises from specialized ependymocytes of the subcommissural organ, which is responsible for the secretion of glycopeptides and is located below the posterior commissure at the level of the cerebral aqueduct, just anterior to the pineal gland. This glycopeptide content is likely responsible for the T1 hyperintensity commonly reported in PTPR [6]. This finding, however, is not pathognomonic of PTPR, but, in a study of 4 patients reported by Chang et al., they demonstrated this characteristic T1 hyperintensity in all cases using conventional T1 SE sequences [2]. Our report confirms that PTPR is a possible diagnosis when a solid, heterogeneous mass is observed, particularly if it is associated with cysts and an intrinsic T1 hyperintensity.

MTC is a useful technique for manipulating tissue contrast on MRI and is based on the difference in magnetic field-induced frequencies of free water protons and macromolecule-bound water protons [7]. In some tissues, such as white and gray matter, the rate of exchange is very high because the macromolecules have numerous surface sites where exchange between the two pools is possible. MTC consists of applying an additional radiofrequency pulse to presaturate macromolecule protons that suppress the signal from the adjacent parenchyma, enhancing the contrast and making pathological changes more clear [8]. Our results suggest that T1 SE MTC is more effective in detecting this peculiar hyperintensity related to PTPR. However, fat content must be excluded because it is most commonly related to teratomas. Melanin, calcification and extracellular methemoglobin, usually seen in melanotic tumors, and hemorrhagic metastases, choriocarcinomas or teratomas, must also be excluded.

Treatment for PTPR has not yet been established due to the small number of reported cases. The treatment of choice is surgery and radiotherapy [9]. The clinical course of PTPR is characterized by frequent local recurrence [10]. The entire neuraxis should be imaged, because leptomeningeal seeding has been documented [2].

The patient is currently at six months after surgery and has showed improvement of symptoms. Thus far, there are no signs of CSF dissemination, with indolent tumor evolution.

## 4. Conclusions

Tumors of the pineal region have broad differential diagnosis. However, PTPR should be suggested for a solid and heterogeneous mass containing cysts located in the posterior commissure or pineal region, particularly if T1 hyperintensity is noticed. Our report supports the inclusion of a T1 SE MTC sequence in the MR protocol to better demonstrate T1 hyperintensity in pineal region tumors, particularly if T1 hyperintensity unrelated to fat, melanin, or microcalcification is noted. Nevertheless definitive diagnosis is provided by immunohistochemical studies.
